# RETRACTED: Markouli et al. Impact of Histone Modifications and Their Therapeutic Targeting in Hematological Malignancies. *Int. J. Mol. Sci.* 2022, *23*, 13657

**DOI:** 10.3390/ijms25073762

**Published:** 2024-03-28

**Authors:** Mariam Markouli, Dimitrios Strepkos, Christina Piperi

**Affiliations:** Department of Biological Chemistry, Medical School, National and Kapodistrian University of Athens, 11527 Athens, Greece; smd1800087@uoa.gr (M.M.); smd1700150@uoa.gr (D.S.)

The *International Journal of Molecular Sciences* Editorial Office retracts the article “Impact of Histone Modifications and Their Therapeutic Targeting in Hematological Malignancies” [1] cited above.

Following publication, the corresponding author contacted the Editorial Office regarding an overlap with a previously published review article [2] by another group of authors. 

Adhering to our complaint’s procedure, an investigation was conducted by the Editorial Office and the Editorial Board that confirmed a significant overlap between the two review articles and a lack of appropriate citation or acknowledgement. Given the extent of the overlap, the Editorial Office and Editorial Board have decided to retract this article as per MDPI’s retraction policy (https://www.mdpi.com/ethics#_bookmark30).

This retraction was approved by the Editor-in-Chief of the journal *International Journal of Molecular Sciences.*

The authors agreed to this retraction.
